# Air Pollution Exposure and Birth Weight in the ECHO Cohort

**DOI:** 10.1001/jamanetworkopen.2025.51459

**Published:** 2025-12-26

**Authors:** Whitney Cowell, Hsiao-Hsien Leon Hsu, Allan C. Just, Itai Kloog, Brent A. Coull, Ander Wilson, Alison E. Hipwell, Margaret R. Karagas, Frank D. Gilliland, Amy M. Padula, Kecia N. Carroll, Jean M. Kerver, Akhgar Ghassabian, Carlos A. Camargo, Dana Dabelea, Daphne Koinis-Mitchell, Viren D’Sa, Mehtap Haktanir Abul, Joseph M. Braun, Lisa A. Croen, Tina Hartert, Akihiro Shiroshita, Janet L. Peacock, Jenae M. Neiderhiser, Leslie D. Leve, Jody M. Ganiban, Augusto A. Litonjua, Cindy T. McEvoy, Meredith B. Haag, Rebecca J. Schmidt, Amanda J. Goodrich, Kristen Lyall, Heather E. Volk, Thomas G. O’Connor, David Q. Rich, Christine A. Porucznik, Rosalind J. Wright

**Affiliations:** 1 Departments of Pediatrics & Population Health, New York University Grossman School of Medicine, New York, New York; 2 Department of Environmental Medicine & Climate Science, Icahn School of Medicine at Mount Sinai, New York, New York; 3 Department of Epidemiology, Brown University School of Public Health, Providence, Rhode Island; 4 Department of Biostatistics, Harvard T.H. Chan School of Public Health, Boston, Massachusetts; 5 Department of Statistics, Colorado State University, Fort Collins; 6 Department of Psychiatry, University of Pittsburgh, Pittsburgh, Pennsylvania; 7 Department of Epidemiology, Geisel School of Medicine at Dartmouth, Dartmouth College, Hanover, New Hampshire; 8 Department of Population and Public Health Sciences, Keck School of Medicine, University of Southern California, Los Angeles; 9 Department of Obstetrics, Gynecology and Reproductive Sciences, Program for Reproductive Health and the Environment, University of California, San Francisco; 10 Department of Pediatrics, Icahn School of Medicine at Mount Sinai, New York, New York; 11 Department of Epidemiology & Biostatistics, College of Human Medicine, Michigan State University, East Lansing; 12 Department of Emergency Medicine, Massachusetts General Hospital, Harvard Medical School, Boston; 13 Lifecourse Epidemiology of Adiposity and Diabetes Center, University of Colorado Anschutz Medical Campus, Aurora; 14 Department of Pediatrics, Rhode Island Hospital and The Warren Alpert Medical School of Brown University, Providence; 15 Division of Pulmonary Medicine, Department of Pediatrics, Brown University School of Medicine, Providence, Rhode Island; 16 Division of Research, Kaiser Permanente Northern California, Pleasanton; 17 Vanderbilt University Medical Center, Medicine, Nashville, Tennessee; 18 Division of Epidemiology, Department of Medicine, Vanderbilt University School of Medicine, Nashville, Tennessee; 19 Department of Psychology, The Pennsylvania State University, University Park; 20 Department of Counseling Psychology and Human Services, University of Oregon, Eugene; 21 Department of Psychological and Brain Sciences, George Washington University, Washington, District of Columbia; 22 Division of Pediatric Pulmonary Medicine, Department of Pediatrics, University of Rochester Medical Center, Rochester, New York; 23 Papé Pediatric Research Institute, Department of Pediatrics, Oregon Health & Science University, Portland; 24 Division of Neonatology, Department of Pediatrics, Oregon Health & Science University, Doernbecher Children’s Hospital, Portland; 25 Department of Public Health Sciences, University of California Davis School of Medicine, Davis; 26 AJ Drexel Autism Institute, Drexel University, Philadelphia, Pennsylvania; 27 Department of Mental Health, Johns Hopkins Bloomberg School of Public Health, Baltimore, Maryland; 28 Departments of Psychiatry, Neuroscience, and Obstetrics and Gynecology, University of Rochester, Rochester, New York; 29 Department of Public Health Sciences, University of Rochester School of Medicine and Dentistry, Rochester, New York; 30 Department of Family and Preventive Medicine, Spencer Fox Eccles School of Medicine, University of Utah, Salt Lake City; 31 Department of Public Health, Icahn School of Medicine at Mount Sinai, New York, New York

## Abstract

**Importance:**

Prior studies report negative associations between prenatal exposure to fine particulate matter (ie, aerodynamic diameter <2.5 µg; PM_2.5_) and birth weight, but have typically averaged exposure across pregnancy, which may not reveal windows of susceptibility.

**Objective:**

To identify windows of prenatal susceptibility to PM_2.5_.

**Design, Setting, and Participants:**

This was a retrospective analysis of a prospectively enrolled cohort study. Participants were enrolled at 1 of 50 sites participating in the US Environmental Influences on Child Health Outcomes Cohort. The study included full-term, singleton births occurring between September 2003 and December 2021. Statistical analyses were conducted from March 2024 to February 2025.

**Exposures:**

Daily residential PM_2.5_ exposure was estimated using a machine-learning model covering the contiguous US and mean exposure estimates were calculated for each week of pregnancy.

**Main Outcomes and Measures:**

Bayesian distributed lag interaction models were used to examine cumulative and week-specific associations between PM_2.5_ exposure and birth weight for gestational age (BWGA) *z* scores. Interactions with sex, race and ethnicity, and region were also examined.

**Results:**

The sample of 16 868 mother-newborn pairs (maternal mean [SD] age, 30.4 [5.5] years; 605 [3.6%] Asian, 2197 [13.0%] Black or Black-Hispanic, 3407 [20.2%] Hispanic, 9251 [54.8%] non-Hispanic White, and 1408 [8.4%] other) included 15 806 unique mothers and 1062 mothers with 2 or more children in the study. Mean (SD) weekly PM_2.5_ exposure during pregnancy was relatively low, at 8.03 (2.3) µg/m^3^, and overall mean (SD) birth weight was 3410.7 (464.5) g. In the sample overall, there was a negative association between PM_2.5_ exposure and BWGA *z* score (β = −0.06; 95% credible interval [CrI], −0.10 to −0.03), with a critical window in early gestation (weeks 1-5) that persisted only among males (β = −0.06; 95% CrI, −0.10 to −0.02). When examining differences by region, there were negative associations in the Northeast (β = −0.09; 95% CrI, −0.15 to −0.03), Midwest (β = −0.11; 95% CrI, −0.17 to −0.05; critical window, 12-18 weeks), and South (β = −0.18; 95% CrI, −0.17 to −0.05; critical window, 3-9 weeks).

**Conclusions and Relevance:**

In this cohort study, higher PM_2.5_ exposure was associated with lower BWGA *z* score, with critical windows identified during early pregnancy to midpregnancy; however, findings varied by sex and region. Understanding windows of susceptibility to environmental exposures can help guide research on underlying biological processes and can inform strategies for limiting exposure during certain periods of pregnancy.

## Key Points

**Question:**

Are there critical windows of prenatal susceptibility to fine particulate matter exposure, and do they vary by geographic and demographic factors?

**Findings:**

This cohort study of 16 868 mother-newborn pairs in the Environmental Influences on Child Health Outcomes Cohort found that greater fine particulate matter exposure was negatively associated with birth weight, with a critical window during early pregnancy. The magnitude of association and windows of susceptibility varied by sex and US region but not race and ethnicity.

**Meaning:**

These findings on windows of susceptibility to environmental exposures can help guide research on underlying developmental mechanisms and can inform strategies for reducing exposure during vulnerable periods.

## Teaser

This cohort study in the Environmental Influences on Child Health Outcomes (ECHO) Cohort examines the association of mean weekly prenatal exposure to fine particulate matter with birth weight for gestational age.

## Introduction

Low birth weight is a risk factor for neonatal mortality and a range of morbidities that extend into later life.^1,2,3^ These complications are not limited to infants born prematurely, but may also affect small infants born at term, suggesting that factors that influence growth independent of gestational age are important determinants of health.^4^ Environmental exposures during pregnancy can disrupt a range of biological processes along the maternal-placental-fetal axis, with potential consequences for perinatal outcomes. Fine particulate matter with an aerodynamic diameter less than 2.5 µm (PM_2.5_) is 1 of 6 criteria air pollutants regulated by the US Environmental Protection Agency (EPA). Animal and in vitro studies suggest that PM_2.5_ exposure leads to placental inflammation,^5,6^ altered placental DNA methylation^7^ and protein expression,^8^ and impaired trophoblast invasion,^9,10^ all of which can perturb maternal-fetal nutrient transfer and influence fetal growth.^11,12,13^ Several epidemiologic studies have reported negative associations between prenatal PM_2.5_ exposure and birth weight.^14,15,16^ However, most studies have used the mean exposure across pregnancy or trimesters, precluding the ability to detect windows of susceptibility that may arise when the effects of an exposure vary by the developmental processes with which it coincides.^17^ Understanding these windows can help guide research on underlying biological processes and can inform strategies for reducing exposure during vulnerable periods.

In this study, we examined weekly exposure to PM_2.5_ across pregnancy in relation to term birth weight for gestational age (BWGA) *z* scores. Our sample included nearly 17 000 participants and their infants from across the contiguous US, with weekly PM_2.5_ concentrations estimated at a high spatial resolution. Given our large, diverse sample with broad geospatial heterogeneity, we explored a series of effect modifiers (sex, race and ethnicity, geographic region). We evaluated whether sex at birth modified associations and susceptible windows, given established sex differences in fetal growth^18^ and perinatal complications.^19,20^ We considered race and ethnicity as a modifier because racial and ethnic inequalities in exposure to PM_2.5_ in the US are well documented, with the burden of pollution disproportionately affecting Black and Hispanic populations.^21,22,23,24^ Racially minoritized groups may also face a disproportionate health burden from PM_2.5_ exposure due to increased biological susceptibility stemming from differences in baseline health status, experiences of heightened psychosocial stress, or reduced access to health care services. Notably, persistent racial and ethnic disparities have also been observed for birth outcomes.^25,26^ We evaluated effect modification by geographic region, as nationwide studies have shown that particulate composition varies across the US due to different emission sources.^27^ We hypothesized that higher prenatal exposure to PM_2.5_ would be associated with lower birth weight and that associations would vary depending on the gestational timing of exposure.

## Methods

### ECHO Cohort

This retrospective analysis included mother-newborn pairs prospectively enrolled in the US-based Environmental Influences on Child Health Outcomes (ECHO) Cohort. All study protocols were approved by cohort-specific and/or central ECHO institutional review boards, and all participants provided written informed consent. This study followed the Strengthening the Reporting of Observational Studies in Epidemiology (STROBE) reporting guideline. We restricted the sample to participants with available information on residential history during pregnancy and only included births occurring between September 2003 and December 2021, as this was the time frame of available PM_2.5_ estimates. We excluded sites contributing fewer than 20 participants to improve model convergence. We also restricted the sample to dyads with a singleton livebirth between 37 and 42 weeks’ gestation with available information on race and ethnicity. These criteria resulted in a final sample of 16 868 dyads derived from 50 study sites, as outlined in eFigure 1 in Supplement 1. The sample included 15 806 unique mothers, of whom 14 815 contributed data from 1 child, 929 contributed data from 2 children, 54 contributed data from 3 children, 7 contributed data from 4 children, and 1 contributed data from 5 children, resulting in 6.3% of the sample (1062 children) representing siblings.

### PM_2.5_ Estimates

We used a daily high-resolution PM_2.5_ machine-learning model covering the contiguous US from 2003 through 2021 to estimate participant residential exposure based on geocoded addresses. The model, referred to as XGBoost-IDW Synthesis (XIS-PM_2.5_),^28^ uses a machine-learning algorithm called extreme gradient boosting in combination with inverse-distance weighting to make predictions at arbitrary points, allowing for address-level exposures to be estimated. Model inputs, prediction modeling, and evaluation have been described in detail elsewhere^28^ and are briefly outlined in the eMethods 1 in Supplement 1. We calculated mean weekly PM_2.5_ concentrations for each participant across pregnancy by using 7-day intervals beginning on gestational day 1. In addition to PM_2.5_, we estimated daily mean ambient residential temperature and used the mean across pregnancy, using a XIS-temperature model that relies on remote sensing in combination with a parsimonious set of variables. The modeling approach parallels that described for XIS-PM_2.5_ and has been described in detail elsewhere.^29^

### Birth Weight

Information on birth weight and gestational age was extracted from the medical record, self-reported, or measured by study staff. We calculated sex-specific BWGA *z* scores using the 2017 US birth weight reference,^30^ which we chose because it reflects nationally representative data on birth weight and gestational age in the US.

### Covariates

Covariates included maternal age (continuous, in years),^31^ education (<high school, high school degree or equivalent, some college or trade school, Bachelor’s degree, or ≥Master’s degree),^32,33^ parity (nulliparous or multiparous),^34^ tobacco use during pregnancy (any or none),^35^ prepregnancy body mass index (BMI; calculated as weight in kilograms divided by height in meters squared),^36^ newborn sex (male or female), mean ambient temperature across pregnancy (continuous, in Celsius),^37,38^ and geographic region as defined by the US Census Bureau (Northeast, South, Midwest, or West). We evaluated the interaction between PM_2.5_ and race and ethnicity but did not otherwise consider race and ethnicity as covariates. Race and ethnicity were grouped into mutually exclusive categories of Asian, Black or Black-Hispanic, Hispanic (all individuals identifying as Hispanic other than Black-Hispanic), non-Hispanic White, or other, which included participants who identified as American Indian or Alaska Native, Native Hawaiian or Other Pacific Islander, or more than 1 race. We grouped individuals who self-identified as Black-Hispanic with those identifying as Black, as the burden of health disparities among Black-Hispanic individuals tends to more closely parallel that of Black individuals in the US, likely reflecting society’s unequal treatment of individuals on the basis of race.^39^ Race and ethnicity information was self-reported by the mother or abstracted from medical records. Information on education, smoking during pregnancy, and parity was missing for 35%, 17%, and 16% of participants, respectively. Missingness in these variables did not vary by mean PM_2.5_ exposure across pregnancy, BWGA *z* score, race and ethnicity, or region. Missing covariate data were multiply imputed using the MICE package (10 iterations, 10 imputed datasets) in R software version 3.6.2 (R Project for Statistical Computing). For continuous variables, we calculated the mean across imputations, and for categorical variables, we used the modal value to construct a single imputed dataset.

### Statistical Analysis

We used separate bayesian distributed lag interaction models (BDLIMs) to examine cumulative and week-specific associations between PM_2.5_ exposure and BWGA *z* scores with interactions for newborn sex, race and ethnicity, and region. Distributed lag models offer a flexible strategy for investigating critical windows by allowing the joint assessment of sequential short intervals (ie, weeks) throughout a specified period (ie, gestation).^40^ The model also estimates the cumulative association, interpreted as the expected change in BWGA *z* score per 1-µg/m^3^ increase in PM_2.5_ at every time point in pregnancy. BDLIMs are an extension of distributed lag models that allow the distributed lag function to differ by a hypothesized effect modifier. The model decomposes the function into a shape component for identifying windows and a scale component for estimating the within-window association, which has the advantage that it allows one, both, or neither to vary by subgroup.^40^ A mathematical description of the model is provided in eMethods 2 in Supplement 1. We evaluated model fit using posterior model probability and deviance information criterion. While all births occurring after 37 weeks were included, the distributed lag function, and hence the evaluation of critical windows, was restricted to the first 37 weeks of pregnancy to allow for equal exposure timing. BDLIMs cannot account for within-family clustering. As an alternative approach to evaluate the influence of including siblings on the results, we ran a sensitivity analysis retaining only the first enrolled child within a sibling set (15 806 children). All statistical analyses were conducted from March 2024 to February 2025 using R version 3.6.2 and BDLIMs were modeled using the *regimes* package.

## Results

### Sample Characteristics

The sample of 16 868 mother-newborn pairs (maternal mean [SD] age, 30.4 [5.5] years; 605 [3.6%] Asian, 2197 [13.0%] Black or Black-Hispanic, 3407 [20.2%] Hispanic, 9251 [54.8%] non-Hispanic White, and 1408 [8.4%] other) included 15 806 unique mothers. There were 2845 mothers (16.7%) with a high school education or less, and 5609 mothers (33.2%) were nulliparous. Most participants resided in the Northeast (5929 [35.2%]), followed by the West (4742 [28.1%]), Midwest (3610 [21.4%]), and South (2587 [15.3%]). Additional descriptive characteristics are provided in the Table. Newborns had a mean (SD) birth weight of 3411 (464) g and gestational age of 39.1 (1.1) weeks; birth weight was similar for males (8702 newborns [51.6%]; mean [SD] birth weight, 3474.2 [474.2] g) and females (8166 newborns [48.4%]; mean [SD] birth weight, 3343.1 [444.1] g) (Table; eFigure 2 in Supplement 1). We observed differences in birth weight by race and ethnicity, with newborns of mothers who self-identified as Black or Black-Hispanic (mean [SD], 3234.1 [452.7] g) or Asian (mean [SD], 3240.5 [404.9] g) smaller than those of mothers who identified as non-Hispanic White (mean [SD], 3470.5 [456.1] g) (Table; eFigure 3 in Supplement 1). We did not observe differences in birth weight by US region (Table; eFigure 3 in Supplement 1).

**Table.** Characteristics of Study Participants and Birth Weight by Categorical Characteristic

Characteristic	Participants, No. (%) (N = 16 868)	Birth weight, mean (SD), g
Residential US geographic region		
Northeast	5929 (35.2)	3392.7 (464.4)
Midwest	3610 (21.4)	3460.6 (464.3)
South	2587 (15.3)	3376.6 (461.3)
West	4742 (28.1)	3413.9 (463.6)
Maternal age, mean (SD), y	30.4 (5.5)	NA
Maternal self-reported race and ethnicity		
Asian	605 (3.6)	3240.5 (404.9)
Black or Black-Hispanic	2197 (13.0)	3234.1 (452.7)
Hispanic^a^	3407 (20.2)	3394.2 (462.8)
Non-Hispanic White	9251 (54.8)	3470.5 (456.1)
Other^b^	1408 (8.4)	3406.7 (474.0)
Maternal education		
<High school	1070 (6.3)	3321.9 (465.6)
High school degree or equivalent	1775 (10.5)	3334.2 (456.9)
Some college or trade school	2327 (13.8)	3396.4 (471.5)
Bachelor’s degree	2991 (17.8)	3445.3 (450.8)
≥Master’s degree	2817 (16.7)	3418.2 (446.0)
Missing	5888 (34.9)	NA
Maternal smoking during pregnancy		
None	13242 (76.1)	3417.3 (461.0)
Any	1159 (6.9)	3326.6 (477.6)
Missing	2862 (17.0)	NA
Maternal prepregnancy BMI, mean (SD)	26.9 (6.9)	NA
Parity		
Nulliparous	5609 (33.2)	3356.6 (453.0)
Multiparous	8496 (50.4)	3444.5 (463.6)
Missing	2763 (16.4)	NA
Newborn sex		
Male	8702 (51.6)	3474.2 (474.2)
Female	8166 (48.4)	3343.1 (444.1)
Overall birth weight	NA	3410.7 (464.5)
Gestational age at birth, median (IQR), wk	39.1 (1.1)	NA
PM_2.5_ across pregnancy, mean (SD), µg/m^3^	8.03 (2.3)	NA
Temperature across pregnancy, median (IQR), ° C	12.2 (4.2)	NA

Abbreviations: BMI, body mass index (calculated as weight in kilograms divided by height in meters squared); PM_2.5_, fine particulate matter (aerodynamic diameter <2.5 µm).

^a^ The Hispanic category included any participant self-identifying as Hispanic other than those who indicated Black-Hispanic race and ethnicity, which were grouped with Black.

^b^ Other was defined as American Indian or Alaska Native, Native Hawaiian or Other Pacific Islander, or more than 1 race.

### PM_2.5_ Exposure

The distribution of PM_2.5_ exposure when the mean was calculated across pregnancy was approximately normal, with a mean (SD) of 8.03 (2.30) µg/m^3^ and a range of 2.38 to 18.78 µg/m^3^. The mean exposure level across pregnancy was below the prior (12.0 µg/m^3^) and current (9.0 µg/m^3^) US EPA National Ambient Air Quality Standard for PM_2.5_. eFigure 4 in Supplement 1 presents mean PM_2.5_ levels across pregnancy by US county of participant address, and eFigure 5 in Supplement 1 displays boxplots of mean PM_2.5_ across pregnancy by study site. We did not observe differences in residential mean PM_2.5_ levels across pregnancy by infant sex (eFigure 6 in Supplement 1); however, PM_2.5_ concentrations did vary by geographic region, with the highest exposure occurring in the South (mean [SD], 9.73 [2.02] µg/m^3^) and the lowest exposure occurring in the Northeast (mean [SD], 6.90 [1.85] µg/m^3^) (eFigure 7 in Supplement 1). PM_2.5_ also varied by race and ethnicity, with the highest exposure among participants who identified as Black or Black-Hispanic (mean [SD], 9.82 [2.42] µg/m^3^) and the lowest among non-Hispanic White participants (mean [SD], 7.54 [2.10] µg/m^3^) (eFigure 7 in Supplement 1). We highlight that the exposure differences observed by region are not entirely explained by demographics, as the racial and ethnic disparity in exposure persisted across the Northeast, Midwest, and South (eFigure 7 in Supplement 1).

### Critical Windows and Interactions With Newborn Sex, Race and Ethnicity, and Geographic Region

**Figure 1.**
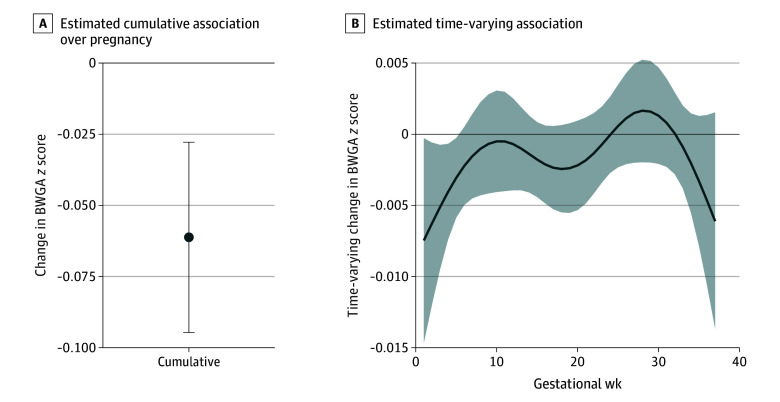
Cumulative and Time-Varying Associations Between Weekly Prenatal Fine Particulate Matter (PM_2.5_) and Birth Weight For Gestational Age (BWGA) *z* Score Estimates are provided per 1-µg/m^3^ increased PM_2.5_ exposure, and the model is adjusted for maternal age, education, prepregnancy body mass index, parity, tobacco use during pregnancy, ambient temperature, and US geographic region of residence. Error bar (A) and shading (B) indicate 95% credible intervals.

Figure 1 shows the time-varying and cumulative association of weekly PM_2.5_ exposure with BWGA *z* score from BDLIMs evaluating the overall sample. Estimates correspond to the change in BWGA z score per 1-µg/m^3^ increase in PM_2.5_. Model fit statistics are provided in eTable 1 in Supplement 1 and effect estimates with 95% credible intervals (CrIs) are provided in eTable 2 in Supplement 1. In the sample overall, we observed a negative cumulative association of PM_2.5_ exposure across pregnancy with BWGA *z* score (β = −0.06; 95% CrI, −0.10 to −0.03), with a critical window identified from gestational weeks 1 to 5 (Figure 1).

**Figure 2.**
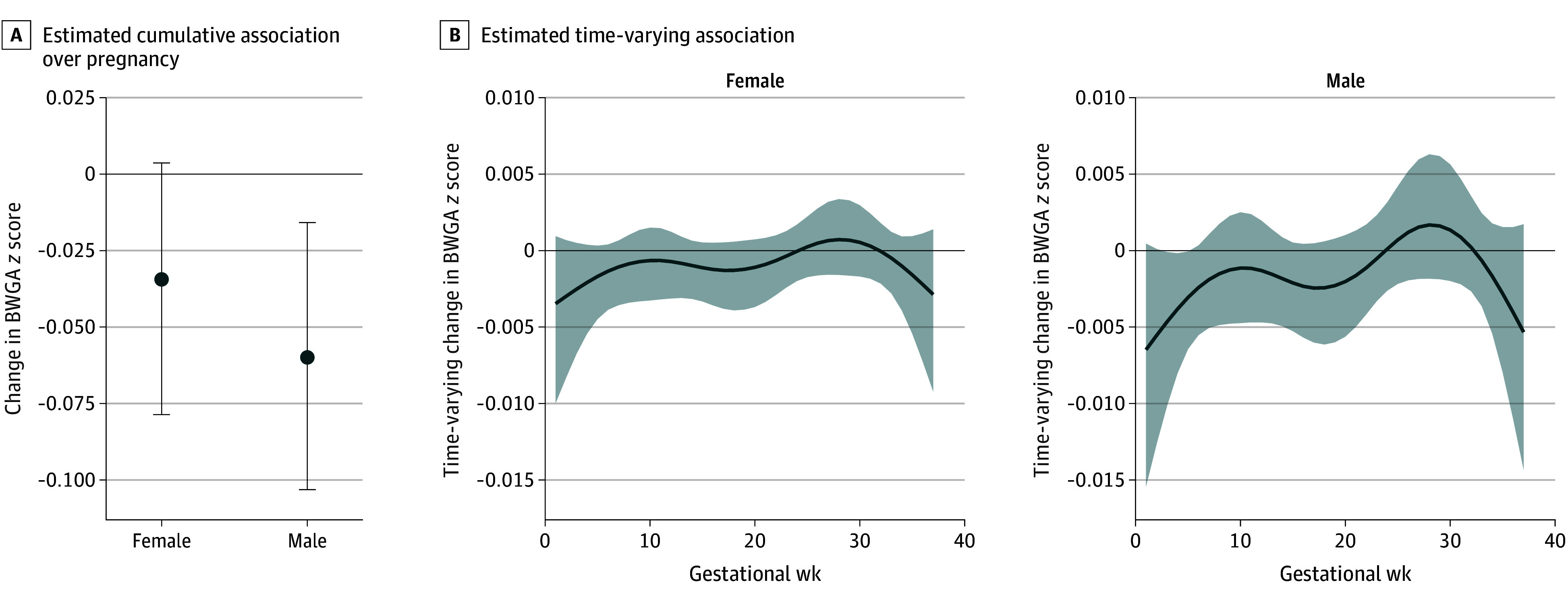
Cumulative and Time-Varying Associations Between Weekly Prenatal Fine Particulate Matter (PM_2.5_) and Birth Weight For Gestational Age (BWGA) *z* Score With an Interaction for Newborn Sex Estimates are provided per 1-µg/m^3^ increased PM_2.5_ exposure, and the model is adjusted for maternal age, education, prepregnancy body mass index, parity, tobacco use during pregnancy, ambient temperature, and US geographic region of residence. Error bars (A) and shading (B) indicate 95% credible intervals.

When examining interactions of sex with PM_2.5_ (Figure 2), the best-fitting model indicated that the weight functions were the same across sexes, while the effect estimates and cumulative associations differed (female: β = −0.03; 95% CrI, −0.08 to 0.00; male: β = −0.06; 95% CrI, −0.10 to −0.02). Although associations were small, a critical window was identified among males from gestational weeks 3 to 5.

**Figure 3.**
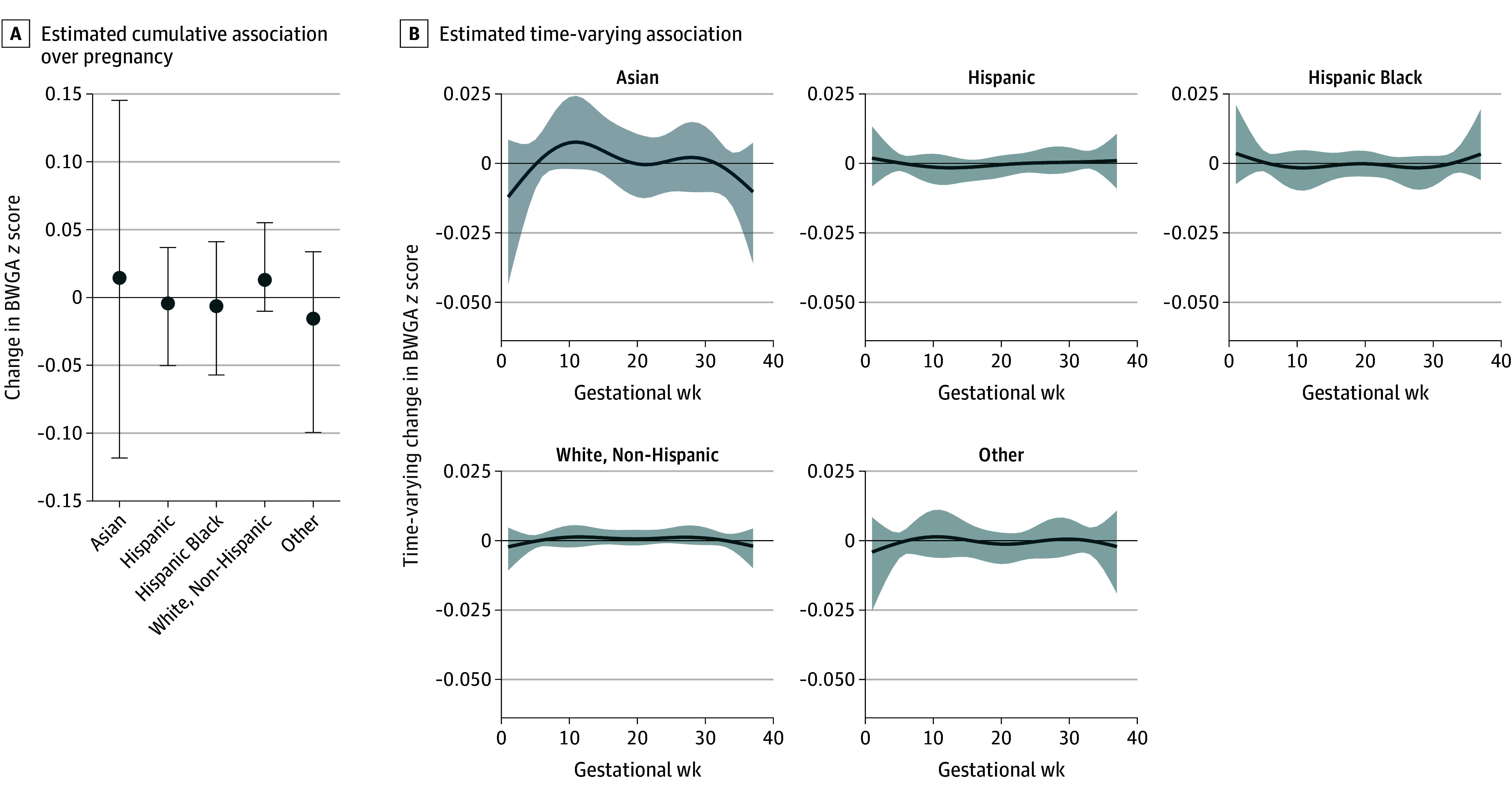
Cumulative and Time-Varying Associations Between Weekly Prenatal Fine Particulate Matter (PM_2.5_) and Birth Weight for Gestational Age (BWGA) *z* Score With an Interaction for Maternal Self-Identified Race and Ethnicity Estimates are provided per 1-µg/m^3^ increased PM_2.5_ exposure, and the model is adjusted for maternal age, education, prepregnancy body mass index, parity, tobacco use during pregnancy, ambient temperature, newborn sex, and US geographic region of residence. Other was defined as American Indian or Alaska Native, Native Hawaiian or Other Pacific Islander, or more than 1 race. Error bars (A) and shading (B) indicate 95% credible intervals.

When examining interactions with race and ethnicity, the best-fitting model suggested small differences in the cumulative association (Asian: β = 0.01; 95% CrI, −0.12 to 0.15; Black or Black-Hispanic: β = −0.01; 95% CrI, −0.06 to 0.04; Hispanic: β = −0.00; 95% CrI, −0.05 to 0.04; non-Hispanic White: β = 0.01; 95% CrI, −0.01 to 0.06; other: β = −0.02; 95% CrI, −0.11 to 0.03). However, time-varying weights across pregnancy were similar, with no critical windows of susceptibility identified (Figure 3).

**Figure 4.**
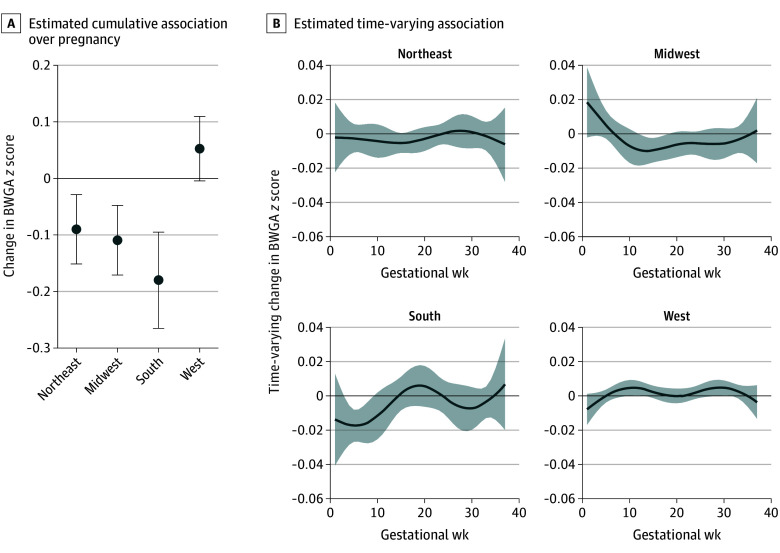
Cumulative and Time-Varying Associations Between Weekly Prenatal Fine Particulate Matter (PM_2.5_) and Birth Weight for Gestational Age (BWGA) *z* Score With an Interaction for US Geographic Region Estimates are provided per 1-µg/m^3^ increased PM_2.5_ exposure, and the model is adjusted for maternal age, education, prepregnancy body mass index, parity, tobacco use during pregnancy, ambient temperature, and newborn sex. Error bars (A) and shading (B) indicate 95% credible intervals.

Finally, when considering the interaction between PM_2.5_ exposure and geographic region, we found different associations across regions and different estimated time-varying weights (Figure 4). In the Northeast, no critical window was observed, but the cumulative association was negative (β = −0.09; 95% CrI, −0.15 to −0.03). In the South and Midwest, cumulative associations were also negative (South: β = −0.18; 95% CrI, −0.17 to −0.10; Midwest: β = −0.11; 95% CrI, −0.17 to −0.05) and critical windows were observed from gestational weeks 3 to 9 and weeks 12 to 18, respectively. In the West, PM_2.5_ showed a small, positive association with BWGA *z* score (β = 0.05; 95% CrI, −0.00 to 0.11), with critical windows identified from gestational weeks 10 to 13 and 29 to 31 (Figure 4).

In the sensitivity analysis retaining only the first enrolled sibling of a sibling set, the cumulative association across pregnancy was similar in magnitude and direction (β = −0.06; 95% CrI, −0.09 to −0.03) compared to the sample overall (β = −0.06; 95% CrI, −0.10 to −0.03), with a more pronounced critical window observed for weeks 1 to 5 and a second window becoming apparent from weeks 16 to 20 (eFigure 8 in Supplement 1).

## Discussion

In this cohort study, we investigated weekly PM_2.5_ exposure across pregnancy in association with BWGA *z* scores in a large, diverse, US-based sample. We found that increased PM_2.5_ exposure was associated with reduced BWGA *z* score, with a critical window during the first 5 weeks of pregnancy. When considering differences by sex, the early window remained only among males. Negative associations also remained in the Northeast, Midwest, and South, but not the West, with critical windows identified from early pregnancy to midpregnancy. In contrast, when examining differences by race and ethnicity, no cumulative associations or critical windows were identified.

Prior studies have examined associations of gestational PM_2.5_ exposure with birth weight^16,41,42,43^; however, limited research has investigated weekly exposure. Consistent with our findings, an analysis of 2328 women enrolled in the Spanish Infancia y Medio Ambiente project found a negative association between PM_2.5_ exposure and late pregnancy estimated fetal weight, with a susceptible window of exposure from gestational weeks 1 to 6 and an overall shape that mirrored the pattern observed in this study.^44^ Two other studies based in Canada^45^ and Massachusetts^46^ with similar exposure levels also found negative associations but identified critical windows of varying lengths in midpregnancy to late pregnancy.^45,46^ Most other studies evaluating critical windows have assigned exposure estimates at the district, census block, or county level using a sparse network of stationary ground monitors^47,48,49,50^ or have examined substantially higher exposure levels (mean ≥50 µg/m^3^).^43,47,48^ Few prior studies have considered sex differences in associations between PM_2.5_ and birth weight, and to our knowledge, none has considered critical windows.^51^

When examining the interaction with race and ethnicity, we observed few differences, which could in part reflect the structure of the ECHO Cohort, whereby the loss of variation when accounting for region in models considering the race and ethnicity interaction limited statistical power to detect differential effects. Although we did not detect an interaction, this does not imply the complete absence of differential vulnerability, which should be explored in future studies designed to investigate disparities in air pollution exposure and birth outcomes.

When examining regional differences in the association between PM_2.5_ and BWGA *z* score, we found negative associations among participants residing in the South and Midwest. Analyses of PM_2.5_ components across the US over the past 25 years have revealed significant spatial variations in chemical constituents.^52,53,54^ This could explain the regional heterogeneity in associations that we observed, as there is growing evidence to show that some PM_2.5_ components are more toxic than others, including with regard to fetal growth.^55,56,57,58,59,60^ Alternatively, the regional findings could reflect differential susceptibility to PM_2.5_ due to social factors, such as access to health care services, that vary regionally across the US.

Using BWGA *z* scores allowed us to capture subclinical differences in newborn weight. While the magnitude of observed associations was modest, the findings carry significance at the population level, given widespread exposure to PM_2.5_ and the potential clinical consequences of decreased birth weight at the lower distribution tail. We also highlight that particulate air pollution exposure is only one of many factors, such as nutrition, psychosocial stress, and access to prenatal care, that influence fetal growth. While a modest decrement in birth weight associated with PM_2.5_ exposure may appear inconsequential, when combined with other environmental factors it may contribute to outcomes with clinical importance, such as low birth weight.

### Strengths and Limitations

A strength of this study is the estimation of PM_2.5_ exposure at a high spatial and temporal resolution, which limited measurement error and allowed us to investigate windows of susceptibility. However, we acknowledge the use of residential location to determine exposure estimates, which does not reflect time-activity patterns, as a limitation. Exposure levels typically occurred at or below the US EPA National Ambient Air Quality Standard for PM_2.5_ of 9.0 µg/m^3^, which was lowered from 12.0 µg/m^3^ in 2024, and thus are reflective of current policies, supporting the generalizability of our findings. This also suggests that even allowable levels of PM_2.5_ exposure may carry risk for health outcomes and emphasizes the importance of revisiting regulatory thresholds. Unfortunately, we did not have information on indoor particulate matter sources or other criteria air pollutants that may covary and interact with PM_2.5_.

One of the primary benefits of studying birth weight is that data are typically accessible and precisely recorded in the medical record. However, birth weight does not directly reflect fetal growth or variability in growth velocity, which could confer differential susceptibility across gestation. Additional strengths of this study include the racially and ethnically diverse sample and the collection of detailed, individual-level information on sociodemographic and behavioral factors associated with adverse birth outcomes; however, as with all epidemiologic studies, unmeasured confounding could have biased results. We acknowledge high rates of missing maternal education, smoking, and parity data as a limitation, which we addressed using multiple imputation.

## Conclusions

In this cohort study, we assessed associations between prenatal exposure to ambient PM_2.5_ and BWGA *z* scores among participants enrolled in the ECHO Cohort. Our study suggests that a stronger association of PM_2.5_ with birth weight may be present in males early in pregnancy and that exposures occurring in the US Northeast, South, and Midwest may have stronger associations with birth weight than those occurring in the West, which could reflect regional differences in particle composition. This study contributes to growing literature examining time-varying PM_2.5_ exposure in relation to birth outcomes.

## Notes

Supplement 1. eMethods 1. Detailed description of PM_2.5_ exposure assessment eMethods 2. Mathematical description of Bayesian distributed lag interaction models eFigure 1. Selection of the analytic study sample eFigure 2. Boxplot of birthweight for gestational age z-score by infant sex eFigure 3. Boxplot of birthweight for gestational age *z*-score by self-reported race/ethnicity and U.S. geographic region eFigure 4. Pregnancy-averaged PM_2.5_ by US county with ≥1 participant enrolled eFigure 5. Boxplots of pregnancy-averaged PM_2.5_ exposure by ECHO study site eFigure 6. Boxplot of pregnancy-averaged PM_2.5_ (µg/m^3^) by infant sex eFigure 7. Boxplot of pregnancy-averaged PM_2.5_ (µg/m^3^) by self-reported race/ethnicity and U.S. geographic region eTable 1. Model fit statistics for associations between PM_2.5_ and BWGA *z*-score with interactions for newborn sex, race/ethnicity, or US geographic region eTable 2. Estimated cumulative association between prenatal PM_2.5_ and birthweight-for-gestational age *z*-score eFigure 8. Cumulative and time-varying associations between weekly prenatal PM_2.5_ and BWGA *z*-score excluding younger siblings in the sample

Supplement 2. Collaborators for the ECHO Cohort Consortium

Supplement 3. Data Sharing Statement
