# Supplementary material for: Air Pollution Exposure and Birth Weight in the ECHO Cohort
Source: JAMA Netw Open. 2025 Dec 26;8(12):e2551459. doi: 10.1001/jamanetworkopen.2025.51459 (PMC12743281; doi:10.1001/jamanetworkopen.2025.51459)
Supplement: Supplement 1. — eMethods 1. Detailed description of PM2.5 exposure assessment eMethods 2. Mathematical description of Bayesian distributed lag interaction models eFigure 1. Selection of the analytic study sample eFigure 2. Boxplot of birthweight for gestational age z-score by infant sex eFigure 3. Boxplot of birthweight for gestational age z-score by self-reported race/ethnicity and U.S. geographic region eFigure 4. Pregnancy-averaged PM2.5 by US county with ≥1 participant enrolled eFigure 5. Boxplots of pregnancy-averaged PM2.5 exposure by ECHO study site eFigure 6. Boxplot of pregnancy-averaged PM2.5 (µg/m3) by infant sex eFigure 7. Boxplot of pregnancy-averaged PM2.5 (µg/m3) by self-reported race/ethnicity and U.S. geographic region eTable 1. Model fit statistics for associations between PM2.5 and BWGA z-score with interactions for newborn sex, race/ethnicity, or US geographic region eTable 2. Estimated cumulative association between prenatal PM2.5 and birthweight-for-gestational age z-score eFigure 8. Cumulative and time-varying associations between weekly prenatal PM2.5 and BWGA z-score excluding younger siblings in the sample [file jamanetwopen-e2551459-s001.pdf]

Supplemental Online Content

Cowell W, Hsu HL, Just AC, et al; ECHO Cohort Consortium. Air pollution exposure and birth weight in the ECHO Cohort. *JAMA Netw Open*. 2025;8(12):e2551459.
doi:10.1001/jamanetworkopen.2025.51459

eMethods 1. Detailed description of $PM_{2.5}$ exposure assessment
eMethods 2. Mathematical description of Bayesian distributed lag interaction models
eFigure 1. Selection of the analytic study sample
eFigure 2. Boxplot of birthweight for gestational age *z*-score by infant sex
eFigure 3. Boxplot of birthweight for gestational age *z*-score by self-reported race/ethnicity and U.S. geographic region
eFigure 4. Pregnancy-averaged $PM_{2.5}$ by US county with ≥1 participant enrolled
eFigure 5. Boxplots of pregnancy-averaged $PM_{2.5}$ exposure by ECHO study site
eFigure 6. Boxplot of pregnancy-averaged $PM_{2.5}$ (μg/m^3) by infant sex
eFigure 7. Boxplot of pregnancy-averaged $PM_{2.5}$ (μg/m^3) by self-reported race/ethnicity and U.S. geographic region
eTable 1. Model fit statistics for associations between $PM_{2.5}$ and BWGA z-score with interactions for newborn sex, race/ethnicity, or US geographic region
eTable 2. Estimated cumulative association between prenatal $PM_{2.5}$ and birthweight-for-gestational age *z*-score
eFigure 8. Cumulative and time-varying associations between weekly prenatal $PM_{2.5}$ and BWGA *z*-score excluding younger siblings in the sample

This supplemental material has been provided by the authors to give readers additional information about their work.

© 2025 Cowell W et al. *JAMA Network Open*.

eMethods 1. Detailed Description of $PM_{2.5}$ Exposure Assessment

Geocoding:
Maternal residential addresses during pregnancy were geocoded using ArcGIS Streetmap Premium (Environmental Systems Research Institute (ESRI), Redlands, CA); residential history was updated to reflect any relocation. We restricted the analysis to high-quality geocoded records defined as the address matching to either a point address location or specific street address.

$PM_{2.5}$ Modeling:
XIS-$PM_{2.5}$ model inputs included longitude and latitude, integer day of the year, an Inverse Distance Weighting (IDW) feature, daily aerosol optical depth (AOD) at 470 nm from the Multi-Angle Implementation of Atmospheric Correction (MAIAC) algorithm for the NASA Aqua and Terra satellites, daily modeled surface $PM_{2.5}$ concentrations from the Modern-Era Retrospective analysis for Research and Applications, Version 2 (MERRA-2), and a parsimonious set of land use, meteorological, and topographical variables. The weighted mean absolute error of predictions (MAE), when averaging across all years from cross validation, indicated excellent model performance. Additional detailed evaluation of XIS-$PM_{2.5}$ predictive performance in site-wise cross-validation, with stratification by year and climate region has been previously published (Just 2025). Compared to the Fused Air Quality Surface Using Downscaling (FAQSD), a leading product from the US Environmental Protection Agency (EPA), XIS-$PM_{2.5}$ has a 17% reduction in MAE.

Just AC, Arfer KB, Rush J, Lyapustin A, Kloog I. XIS-PM(2.5): A daily spatiotemporal machine-learning model for PM(2.5) in the contiguous United States. *Environ Res*. Apr 15 2025;271:120948. doi:10.1016/j.envres.2025.120948

© 2025 Cowell W et al. *JAMA Network Open*.

eMethods 2. Mathematical description of the Bayesian distributed lag interaction model

The Bayesian distributed lag interaction models (BDLIM) model is

$$Y_i = a_j + \beta_j \sum_{t=1}^{37} \omega_{jt} x_{it} + \mathbf{z}_i' \boldsymbol{\gamma} + \varepsilon_i,$$

where:

- Y_i is a continuous response,
- a_j is a fixed subgroup-specific intercept,
- β_j is the regression coefficient characterizing the subgroup-specific association between weighted pollution exposure and children's asthma status,
- $\sum_{t=1}^{37} \omega_{jt} x_{it}$ is the weighted exposure, and
- $\mathbf{z}_i' \boldsymbol{\gamma}$ is the covariate regression term.

The weights (ω_{jt}s) identify variation in the exposure effect over time, and β_j identifies the total effect of the weighted exposure for group j. When weights are constant over time, BDLIM is equivalent to using pregnancy average exposure in a linear regression model. However, when the weight varies by time the model assigns greater relative weight to some periods. These time periods with greater weight are identified as the critical windows. We estimated separate BDLIM models sex, race/ethnicity, or region as subgroups.

BDLIM allows for 4 potential patterns of effect modification by allowing β_j and/or the weights (ω_{jt}s) to be specific to subgroups of the effect modifier or the same for all groups. The 4 patterns of heterogeneity are:

1) Subgroups have different critical windows identified during gestation (e.g., shifted by a few weeks) as represented by different weight functions for each group and the association between weighted exposure and outcome is also different for each subgroup.
2) Subgroups have different critical windows but the same association between exposure and outcome for all subgroups (β_j is the same for all groups)
3) Subgroups have the same critical window (same weight function for all groups) but different association between exposure and outcome for each subgroup
4) Subgroups have the same critical window and the same association between exposure and children's asthma status within the window (no modification).

We quantified the likelihood of each pattern of heterogeneity and estimates the association between exposure and outcome under each of the four effect modification patterns. We proceeded with the pattern that was best supported by the data.

We quantified the likelihood of each pattern of heterogeneity and estimated the association between exposure and outcome under each of the four effect modification patterns. We proceeded with the pattern that was best supported by the data. This Bayesian model places priors on all model parameters and performs inference based on samples from posterior distributions obtained from a Markov chain Monte Carlo (MCMC) fitting algorithm. To ensure rigor, reproducibility, and statistical stability, we doubled the typically recommended number of iterations in our analysis, conducting 200,000 iterations with a 100,000 burn-in. As previously described (Wilson 2017), when a group-specific association varies in only size or window, BDLIMs are more parsimonious, and therefore more powerful in detecting associations, than an approach that applies a standard DLM stratified by each subgroup separately.

Wilson A, Chiu YM, Hsu HL, Wright RO, Wright RJ, Coull BA. Bayesian distributed lag interaction models to identify perinatal windows of vulnerability in children's health. *Biostatistics*. Jul 1 2017;18(3):537-552. doi:10.1093/biostatistics/kxx002

© 2025 Cowell W et al. *JAMA Network Open*.

eFigure 1. Selection of the analytic study sample

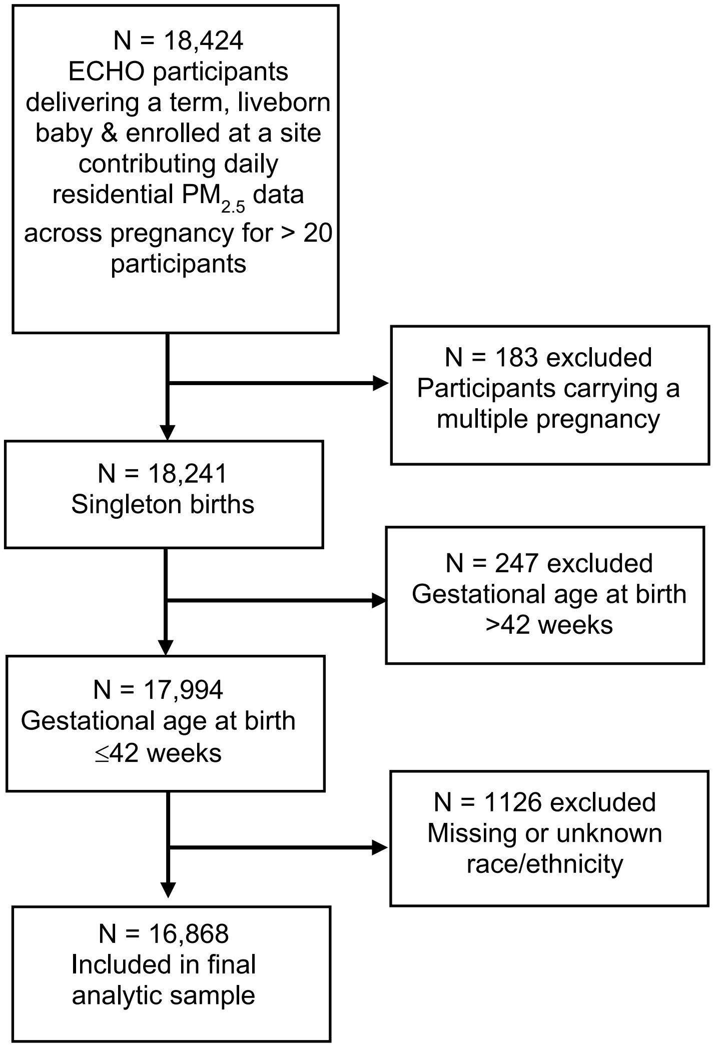

© 2025 Cowell W et al. *JAMA Network Open*.

eFigure 2. Boxplot of birthweight (grams) for gestational age z-score by infant sex.

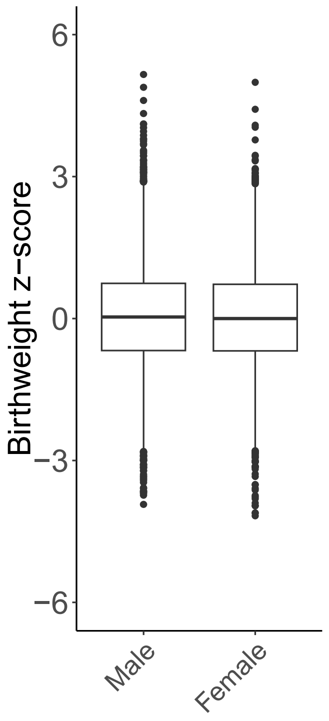

Kruskal-Wallis chi-squared = 1.1333, df = 1, p-value = 0.29

© 2025 Cowell W et al. *JAMA Network Open*.

eFigure 3. Boxplot of birthweight (grams) for gestational age z-score by self-reported race/ethnicity and U.S. geographic region

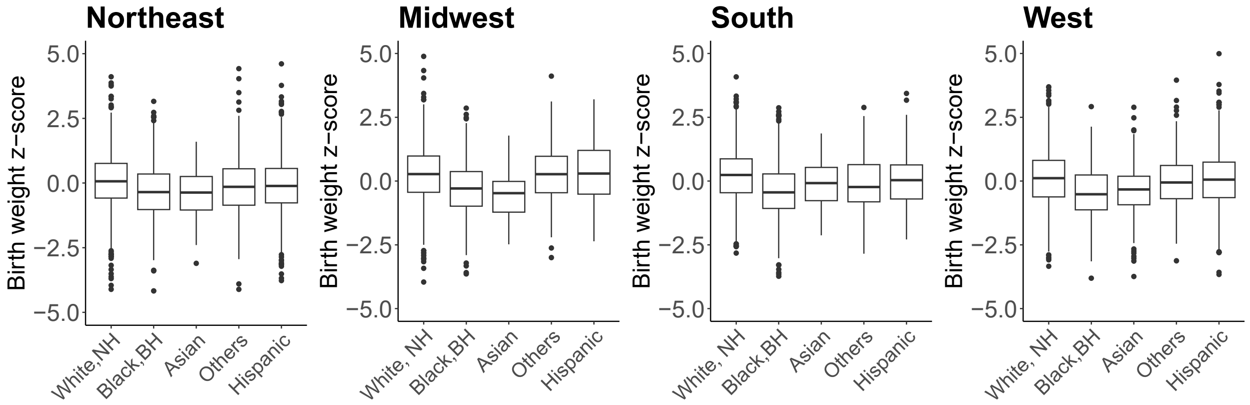

Abbreviations: BH, Black-Hispanic; NH, non-Hispanic.
Black race/ethnicity includes participants who self-identified as Black-Hispanic. Other includes participants who self-identified as American Indian or Alaska Native, Native Hawaiian or other Pacific Islander, or more than one race.

© 2025 Cowell W et al. *JAMA Network Open*.

eFigure 4. Pregnancy-averaged $PM_{2.5}$ by US county with ≥1 participant enrolled

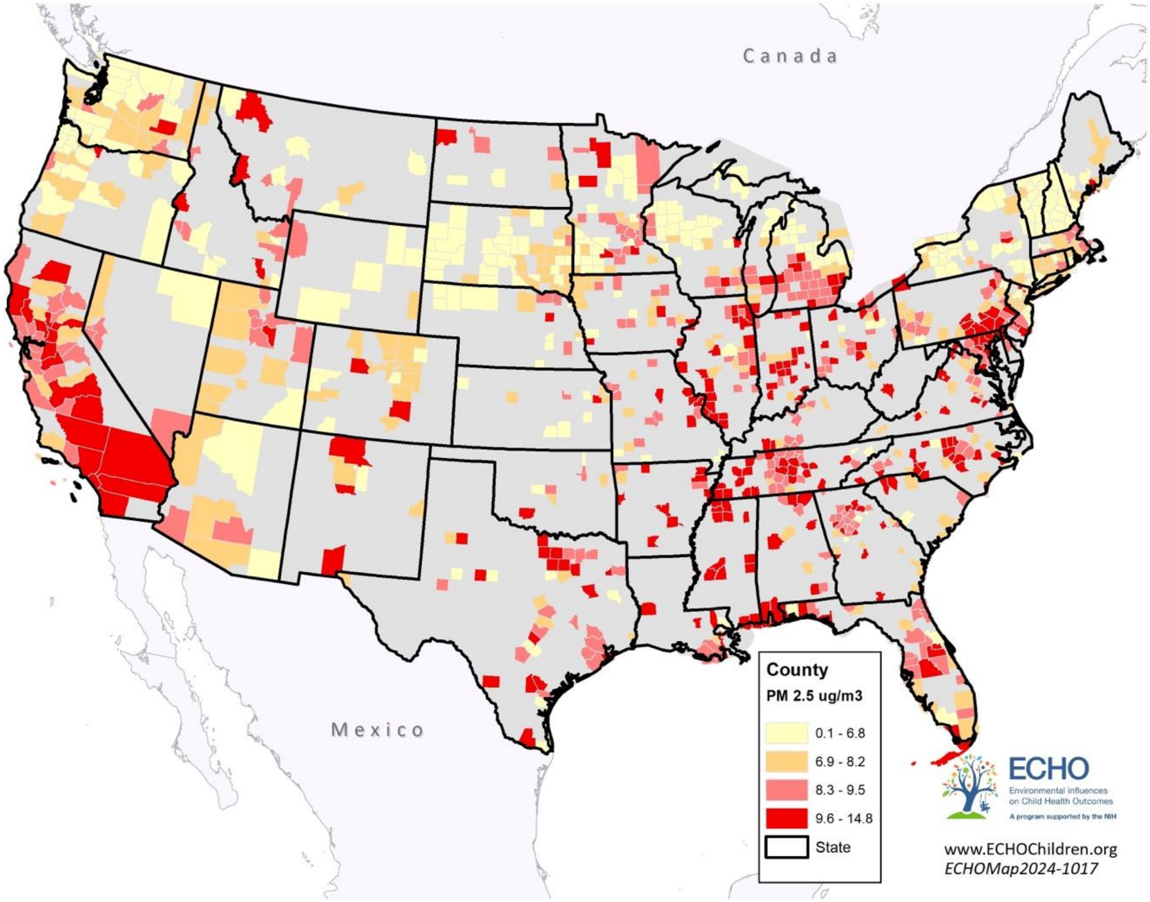

Note: Shading reflects quartile breaks in the $PM_{2.5}$ exposure distribution, which is for visual representation only.

© 2025 Cowell W et al. *JAMA Network Open*.

eFigure 5. Boxplots of pregnancy-averaged $PM_{2.5}$ exposure by ECHO study site

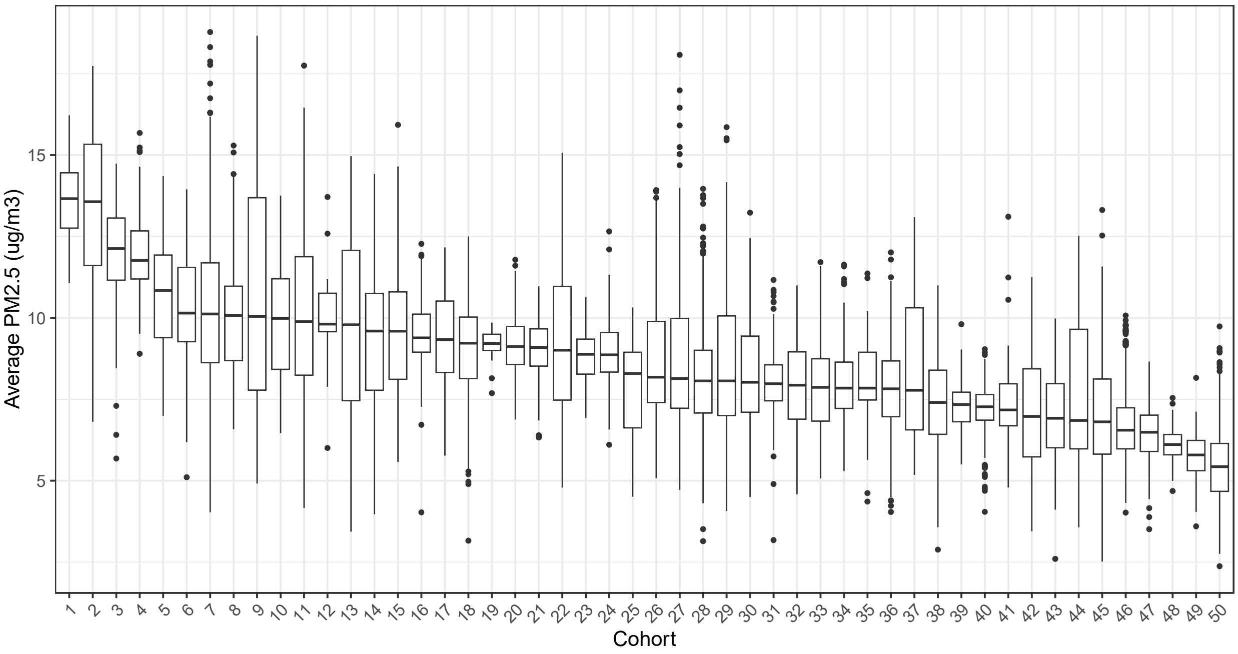

© 2025 Cowell W et al. *JAMA Network Open*.

eFigure 6. Boxplots of pregnancy-averaged $PM_{2.5}$ (μg/m^3) by infant sex

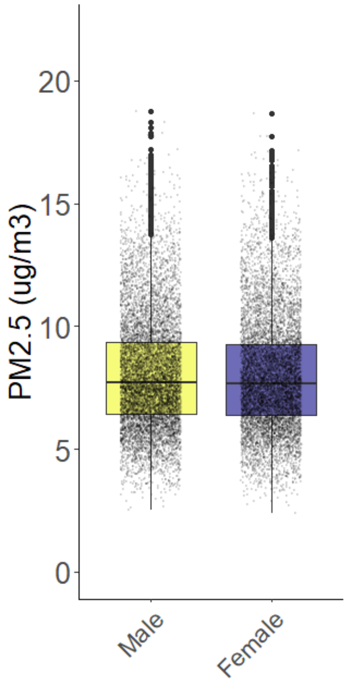

© 2025 Cowell W et al. *JAMA Network Open*.

eFigure 7. Boxplots of pregnancy-averaged $PM_{2.5}$ ($\mu g/m^3$) by self-reported race/ethnicity and U.S. geographic region

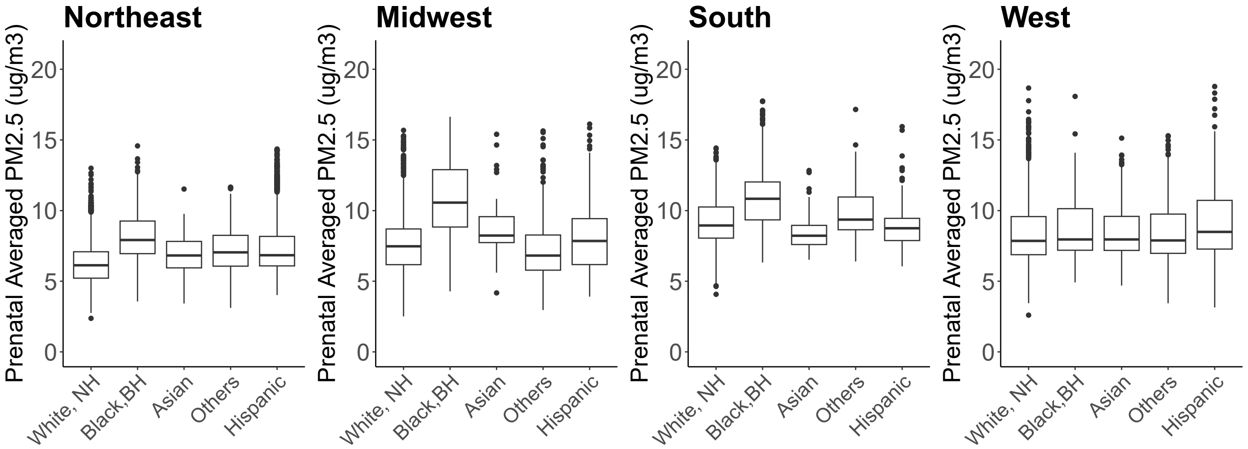

Abbreviations: BH, Black-Hispanic; NH, non-Hispanic.
Black race/ethnicity includes participants who self-identified as Black-Hispanic. Other includes participants who self-identified as American Indian or Alaska Native, Native Hawaiian or other Pacific Islander, or more than one race.

© 2025 Cowell W et al. *JAMA Network Open*.

eTable 1. Model fit statistics for associations between $PM_{2.5}$ and BWGA z-score overall and with interactions for newborn sex, race/ethnicity, or U.S. geographic region

	DIC	pD	Model prob
Overall model	49561.22	19.961	1.00
Newborn sex			
No interaction	49528.71	19.961	0.284
**Different effect size**	**49528.11**	**20.060**	**0.566**
Different windows	49530.07	20.027	0.078
Different effect size and windows	49535.09	24.980	0.073
U.S. geographic region			
No interaction	49528.72	19.968	0.000
Different effect size	49506.07	23.220	0.012
Different windows	49509.16	24.740	0.002
**Different effect size and windows**	**49513.48**	**35.065**	**0.986**
Race/ethnicity			
No interaction	49093.52	23.967	0.007
**Different effect size**	**49093.80**	**28.882**	**0.678**
Different windows	49087.89	21.969	0.250
Different effect size and windows	49111.25	43.996	0.066

For each interaction that was evaluated, the best fitting model is indicated in bold.

© 2025 Cowell W et al. *JAMA Network Open*.

eTable 2. Estimated cumulative association between prenatal $PM_{2.5}$ and birthweight-for-gestational age z-score

	Beta (95% CrI)
Overall	-0.06 (-0.10, -0.03)
Sex	
Female	-0.03 (-0.08, 0.00)
Male	-0.06 (-0.10, -0.02)
Race/ethnicity	
Asian	0.01 (-0.12, 0.15)
Black, Black Hispanic	-0.01 (-0.06, 0.04)
Hispanic	-0.00 (-0.05, 0.04)
White, non-Hispanic	0.01 (-0.01, 0.06)
Other[a]	-0.02 (-0.11, 0.03)
U.S. geographic region	
Northeast	-0.09 (-0.15, -0.03)
Midwest	-0.11 (-0.17, -0.05)
South	-0.18 (-0.17, -0.10)
West	0.05 (-0.00, 0.11)

[a]Other defined as Native Hawaiian or other Pacific Islander, American Indian or Alaska Native, or more than one race.

© 2025 Cowell W et al. *JAMA Network Open*.

eFigure 8. Cumulative and time-varying associations between weekly prenatal $PM_{2.5}$ and BWGA z-score excluding younger siblings in the sample (n=15,806 included)

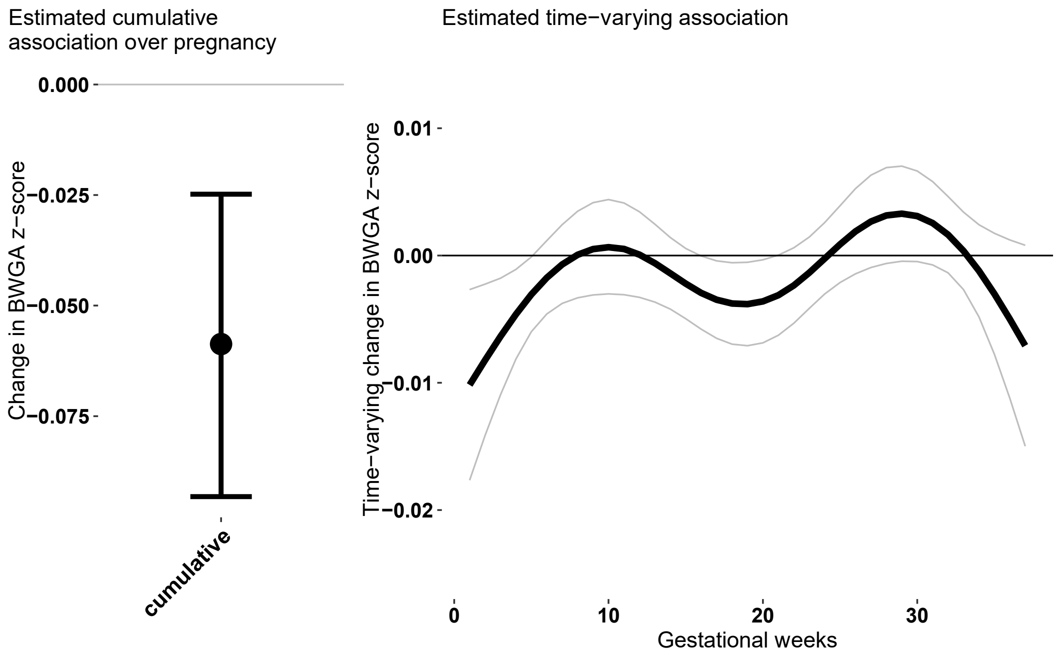

Abbreviations: BWGA, birthweight for gestational age.
Model is adjusted for maternal age, education, pre-pregnancy body mass index, parity, tobacco use during pregnancy, and US geographic region of residence.

© 2025 Cowell W et al. *JAMA Network Open*.
