# Supplementary material for: Air Pollution Exposure and Birth Weight in the ECHO Cohort
Source: JAMA Netw Open. 2025 Dec 26;8(12):e2551459. doi: 10.1001/jamanetworkopen.2025.51459 (PMC12743281; doi:10.1001/jamanetworkopen.2025.51459)
Supplement: Supplement 3. — Data Sharing Statement [file jamanetwopen-e2551459-s003.pdf]

Data Sharing Statement

Cowell. Air Pollution Exposure and Birth Weight in the ECHO Cohort. *JAMA Netw Open*. Published December 26, 2025. doi:10.1001/jamanetworkopen.2025.51459

Data
Data available: No

Additional Information
Explanation for why data not available: Deidentified data are available through NIH DASH online repository, however, participant address information will not be shared to protect privacy.
